# Management of pott’s puffy tumor: a Delphi-method international clinical consensus statement

**DOI:** 10.1007/s00405-025-09589-1

**Published:** 2025-08-09

**Authors:** Nicolien van der Poel, Gopi Shah, Nikolaus Wolter, Christos Georgalas, Anneclaire Vroegop, Vincent Couloigner, Francois Simon, Amanda L. Stapleton, Dana Crosby, Uma Ramaswamy, Patrick Walz, Randa Al-Barazi, Peter-John Wormald, Enrique Ortiz, Perla Villamor, Jin-Young Min, Alvaro Pacheco, Cristobal Langdon, Paresh Naik, Shazia Peer, Rania Mehanna, John R. Craig, Alberto Maria Saibene

**Affiliations:** 1 Department of Otorhinolaryngology, Antwerp University Hospital, Antwerp, Belgium; 2 Faculty of Medicine and Translational Neurosciences, University of Antwerp, Antwerp, Belgium; 3 Pediatric otolaryngologist at ENT Surgery Solutions, Evansville, IN USA; 4 Department of Otolaryngology - Head & Neck Surgery, Hospital for Sick Children, University of Toronto, Toronto, ON Canada; 5 Department of Head, Neck and Skull Base Surgery, Hygeia Hospital, Athens, Greece; 6 Department of Pediatric Otolaryngology, AP-HP, Hôpital Necker Enfants Malades, Paris, France; 7 Université Paris Cité, Paris, F-75006 France; 8 Department of Otolaryngology-Head and Neck Surgery, University of Pittsburgh Medical Center, Children’s Hospital of Pittsburgh of UPMC, Pittsburgh, PA USA; 9 Department of Otolaryngology Head and Neck Surgery, Southern Illinois University School of Medicine, Springfield, IL USA; 10 Department of Otolaryngology-Head and Neck Surgery, University of Pittsburgh Medical Center, Pittsburgh, USA; 11 Department of Pediatric Otolaryngology-Head and Neck Surgery, Nationwide Children’s Hospital, Columbus, OH USA; 12 Department of Otolaryngology and Head and Neck Surgery, American University of Beirut Medical Center, Beirut, Lebanon; 13 Department of Surgery-Otorhinolaryngology Head and Neck Surgery, Central Adelaide Local Health Network, University of Adelaide, Adelaide, SA Australia; 14 Antiguo Hospital Civil de Guadalajara Fray Antonio Alcalde, Guadalajara, Jalisco Mexico; 15 Ear, Nose and Throat Department, American Hospital, Dubai, United Arab Emirates; 16 Department of Otorhinolaryngology-Head & Neck Surgery, Kyung Hee University Hospital, Kyung Hee University College of Medicine, Seoul, Republic of Korea; 17 Clínica Universidad de Los Andes, Santiago, Chile; 18 Department of Pediatric Otorhinolaryngology, Hospital Sant Joan de Deu, Barcelona, Spain; 19 Department of Otorhinolaryngology, Jaslok Hospital & Research Centre, Mumbai, India; 20 Department of Otolaryngology, University of Cape Town, Cape Town, South Africa; 21 Department of Otolaryngology, Our Lady’s Hospital for Sick Children, Crumlin, Dublin, Ireland; 22 Department of Otolaryngology-Head and Neck Surgery, Henry Ford Health, Detroit, MI USA; 23 Otolaryngology Unit, Department of Health Sciences, Santi Paolo e Carlo Hospital, Università Degli Studi di Milano, Milan, Italy

**Keywords:** Pott’s puffy tumor, Acute sinusitis, Intracranial abscess, Pediatric otolaryngology, Expert consensus, Delphi consensus, Clinical guideline

## Abstract

**Purpose:**

Pott’s puffy tumor (PPT) is a rare and complex condition that requires a comprehensive diagnostic approach and multi-faceted treatment strategy. It is associated with a significant risk of intracranial complications. The purpose of this clinical consensus statement (CCS) is to systematically assess diagnostic and therapeutic approaches of patients with PPT, using the best available evidence and expertise of the panel. The results and recommendations are intended to support clinicians in making informed decisions when managing patients with PPT and to standardize diagnostic, antibiotic, and surgical management across institutions. This consensus also aimed to provide a basis for a subsequent international prospective registry to inform data-driven care.

**Methods:**

A literature review was performed via PubMed, and an international panel of 23 experts judged 33 statements in two rounds using a modified Delphi method survey to establish expert recommendations on the diagnostic considerations, medical and surgical management, and postoperative considerations. Strong consensus was defined by a mean score of ≥ 8.00 with no outliers, and consensus by a mean score of ≥ 7.00 with no more than 1 outlier.

**Results:**

A strong consensus was reached on important aspects of the diagnosis and treatment of patients with PPT. Most pivotal are the importance of urgent imaging, imaging of the brain with a preference of MRI, the importance of broad spectrum intravenous antibiotic treatment, and the goal of surgery - resolution of the periosteal abscess and clearance of the frontal sinus drainage pathway - which can be established in various ways and is case and surgeon dependent.

**Conclusion:**

In patients with PPT, a high index of suspicion of intracranial complications is important to prevent any delay in treatment initiation. The recommendations formulated in this international consensus statement aim to improve the diagnosis and care for patients with PPT and to address gaps and uncertainties in current guidelines. Items that did not reach consensus may serve as areas for further research.

**Supplementary Information:**

The online version contains supplementary material available at 10.1007/s00405-025-09589-1.

## Introduction

Pott’s puffy tumor (PPT) is a rare and complex condition that requires a comprehensive diagnostic approach and multi-faceted treatment strategy. PPT is characterized by osteomyelitis of the frontal bone with subperiosteal abscess formation, presenting as localized swelling of the forehead. It is most frequently caused by acute rhinosinusitis, but it can also be caused by chronic rhinosinusitis, trauma to the frontal bone, odontogenic sinusitis, or dental disease [1–3]. Although acute and chronic rhinosinusitis are common in the general pediatric and adult population, PPT remains a rare complication [4]. The incidence of intracranial complications of PPT is significant, and early diagnosis followed by adequate and prompt treatment is mandatory to prevent any long-term sequelae [5]– [6]. PPT was first described by the surgeon Percivall Pott in the 18th century [7]. Since then, over 200 papers have been published, but most of them are case reports, case series, or retrospective in design, as reported in a recent literature review [3]. In the last decades, a variety of treatment options became available: antibiotic therapy, external drainage, endoscopic sinus surgery, and combined approaches [8]. PPT might present with swelling and pain of the forehead and fever, which are usually nonspecific and responsible for a lag in diagnosis. Currently, standardized guidelines guiding healthcare professionals toward the most suitable diagnosis and treatment options for patients presenting with PPT are lacking. This knowledge gap is especially clear in the pediatric space, where decision-making can be especially nuanced. Since the low prevalence of this condition and the lack of high-quality prospective data, we aim to combine the expertise of an international panel with the best available evidence. This consensus survey thereby aims to offer guidance for the diagnostic work-up and therapeutic approach to children and adults with PPT to improve outcomes and patient care.

## Methods

The CCS was developed according to the modified Delphi protocol proposed by Rosenfeld et al. [9].

### Literature review

We performed a systematic literature review (SR) in the MEDLINE database via PubMed. The initial search query was: (“Pott Puffy Tumor“[Mesh]) OR (“Pott Puffy Tumor”). The search strategy adopted was used on July 9th, 2024, and 161 articles were identified. According to the recommendations of Rosenfeld et al. [9], the inclusion of articles should be limited to randomized-controlled trials, guidelines, and SRs. However, since most of the publications about PPTs are case reports, retrospective case series, and literature reviews, we decided to include large case series of ≥ 10 patients and SR. Articles were included when published in English, Italian, German, French, or Spanish. One hundred fifty-one articles were excluded because they were case reports or small retrospective series (< 10 patients), or they did not include PPT patients. Ten articles were eligible for inclusion: five SR and five retrospective series. The reference lists of the SR were hand-checked for further potential missed studies and to highlight the inclusion of articles already identified in the database search in order to avoid data duplication. All activities related to the SR were carried out independently by two members of the development group (NP, GP), with any disagreements resolved through consensus. The final list of included articles—although not subjected to a formal quality appraisal due to the limited amount of available evidence—was nonetheless shared and discussed within the entire development group until a unanimous consensus was reached. The list of selected articles with data extraction is included in the Supplementary Material.

### Panelists’ selection

**Fig. 1:**
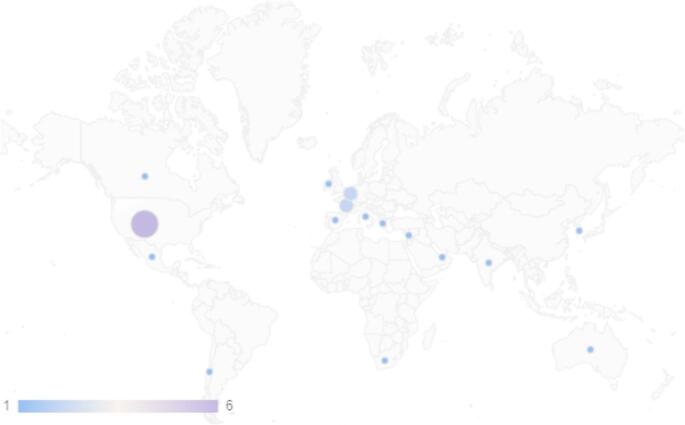
World map reporting the geographic distribution of the panelists

The development group consisted of a chair (NVDP), an assistant chair (GS), and a methodologist (AMS). It included board-certified otolaryngologists with expertise in pediatric and adult sinus surgery, clinical research, and consensus methodology. The panelists were recruited from different international societies and selected based on their clinical, scientific, and educational interests in the subject. All authors have proven clinical expertise in PPT management and/or a record of publications or presentations on the subject. Figure 1 shows the geographic distribution of the panelists.

### Clinical statement development

The development group compiled the initial 33-statement list based on the literature review and a personal assessment of relevant clinical scenarios and potential management issues. The 33 statements were subdivided into the following sections: diagnostic considerations, therapeutic considerations, and follow-up considerations. All panelists were contacted personally by email, and the survey was developed and distributed to the authors via Google Forms (Google LLC, Mountain View, CA, USA). The use of Google Forms was selected based on the prior experience of the study methodologist, as well as for its ease of use, accessibility, and practical advantages. Specifically, it does not require a paid premium version, allows panelists to submit their responses without the need to register a personal account, and automatically generates summary tables that facilitate data analysis and visualization. All of the panelists were instructed to complete the survey anonymously. An independent collaborator (JM) contacted each author and provided a randomly generated personal identification number (PIN) to enter the survey while keeping anonymity. Each of the panelists reported their level of agreement with a 9-point Likert scale (from strongly disagree 1 to strongly agree 9) for each statement, with the option of voicing any comments after each statement. According to the development manual by Ronselfed et al., we defined the results for each statement as follows [9]:

- Strong consensus = mean score of ≥8.00 with no outliers (defined as any rating 2 or more Likert points from the mean in either direction);
- Consensus = mean score of ≥7.00 with no more than 1 outlier;
- Near consensus = mean score of ≥6.50 with no more than 2 outliers;
- No consensus = all other statements.

All authors were encouraged to participate actively via email from creating the initial statements and throughout the entire Delphi consensus process. The number of Delphi rounds was not pre-defined, nor were the rules for revising or excluding statements. Such a decision was made for each non-consensus or near-consensus statement by the development group according to the overall score, outliers, and comments provided by panelists. The first and second Delphi rounds were conducted one month apart, to allow panelists to adequately ponder statements and review them critically for language and content.

## Results

Twenty-five experts participated in the first Delphi round. In the second round, twenty-three experts from sixteen different countries participated. Of the 23 experts, 10 were rhinologists, 11 were pediatric otolaryngologists with special clinical and scientific interest in rhinology, and 2 panelists were double fellowship trained in rhinology and pediatric otolaryngology. The panel included citizens of 16 European, North American, Middle Eastern, and Asian countries, ensuring a rich mix of clinical outlooks.

Of the 33 statements of the first Delphi round, three statements reached a strong consensus, and four statements reached a consensus. The remaining statements were removed, rephrased, or merged according to the scores and anonymous comments of the panelists. The second round consisted of nineteen statements, of which five reached a strong consensus and five reached a consensus. Five statements reached near consensus in the second round, and four statements did not reach consensus. The development group decided not to proceed to a third Delphi round, given the heterogeneous and anonymous comments about the statements reaching near consensus.

The panel reached the highest level of agreement for statements concerning diagnostic considerations, including the role of imaging. The highest scoring statement was “due to the high incidence of intracranial complications associated with PPT, brain imaging is indicated as part of initial work-up, either as CT with contrast or MRI” (mean 8.9, median 9, no outliers). Conversely, topics not reaching consensus were associated with the role of systemic corticosteroids, the use of endonasal stents, and the necessity of frontal sinus obliteration. The lowest scoring statement was “Systemic corticosteroids are not recommended for patients with uncomplicated PPT, but may be indicated in patients with intracranial involvement, only in accordance with neurosurgeons” (mean 7.1, median 8, outliers 6).

The final version of the statements can be found in Tables 1, 2, 3 and 4 (respectively, strong consensus, consensus, near consensus, and no consensus items), further subdivided per section: diagnostic, therapeutic and follow-up considerations. Considering the initial 33 statements, 8 reached a strong consensus (24,2%), 9 reached consensus (27,3%), 5 reached near consensus (15,2%), and 3 reached no consensus (9,1%). The remaining 8 items (24,2%) were merged for content into the other items.

**Table 1:** Statements from the Delphi process reaching strong consensus

Statements from the Delphi process reaching strong consensus	Mean	Median	Outliers
Diagnostic considerations			
In patients with suspected PPT, imaging should be obtained urgently (at least one modality of imaging)	8,8	9	0
Due to the high incidence of intracranial complications associated with PPT, brain imaging is indicated as part of initial work-up, either as CT with contrast or MRI	8,9	9	0
A brain MRI might complement CT scan findings in case of suspected intracranial extension and/or presence of neurological signs and symptoms because it can pick up intracranial complications the CT with contrast could have missed.	8,8	9	0
Patients with PPT should be treated by a multidisciplinary team (otolaryngologist, infectious disease specialist, neurosurgeon in case of intracranial involvement, and pediatrician in patients < 18 years old) for decision-making, management, and follow-up	8,7	9	0
Neurosurgical advice should be sought for patients with PPT with suspected intracranial complications at clinical examination or on imaging	8,8	9	0
***Therapeutic considerations***			
Microbiology samples from the infected sinus or from the abscess should be performed whenever possible (either in the diagnostic phase or during surgery) in patients with suspected/confirmed PPT	8,8	9	0
The choice of antibiotics might be dependent on local, regional, and national guidelines and resistance patterns but should be broad-spectrum covering gram-positives and anaerobes, and should be able to penetrate the blood-brain barrier in case of intracranial involvement	8,7	9	0
The initial surgical approach for treatment of PPT can include incision and drainage of the subperiosteal abscess, endoscopic sinus surgery, trephination, or a combination of these approaches and will depend on the patient’s age, abscess location, surgeon’s preference and experience, and locally available instrumentation and resources.	8,7	9	0

**Table 2:** Statements from the Delphi process reaching consensus

Statements from the Delphi process reaching consensus	Mean	Median	Outliers
Diagnostic considerations			
Patients with suspected PPT should have laboratory investigations, including at least a complete blood count (CBC) with differential	8,5	9	1
In patients suspected with Pott’s Puffy Tumor, a Head CT with contrast should be obtained	8,3	9	1
***Therapeutic considerations***			
Patients with proven Pott’s Puffy Tumor should be admitted for intravenous antibiotics	8,4	9	1
On the basis of the results of the culture, antibiotics should be changed to targeted therapy.	8,4	9	1
The length of iv antibiotics in patients with PPT should be tailored to pathogen, presence of intracranial complications, and degree of osteomyelitic involvement, and it’s better discussed multidisciplinary with an infectious disease specialist	8,6	9	1
The goal of surgery is to drain both the subperiosteal abscess and the sinuses, including the frontal sinus	8,2	9	1
Placement of an external drain should be considered after external drainage/trephination in case of insufficient endonasal drainage of the frontal sinus	8,2	9	1
In surgically treated PPT patients with endoscopic or radiological evidence of inadequate sinus drainage on follow-up, an elective ESS should be considered in case of persisting symptoms	8,5	9	1
As some PPT cases represent a complication of odontogenic sinusitis, such a differential etiology should be considered and, in suspect cases, evaluated with a dental provider	8,2	9	1

**Table 3:** Statements from the Delphi process reaching near consensus

Statements from the Delphi process reaching near consensus	Mean	Median	Outliers
Therapeutic considerations			
Subperiosteal abscess of the frontal bone might require an external approach for drainage in selected cases	8,2	9	2
Patients with PPT without intracranial complications who are not improving with antibiotic treatment should undergo surgical drainage, ideally within 24 h	7,9	8	2
The goal of frontal sinus drainage is to clear purulent material and to provide a drainage pathway in an atraumatic fashion.	8,3	8	2
***Follow-up considerations***			
Short-term endoscopic follow-up is necessary to confirm adequate drainage of the sinuses and infection resolution (2- weeks after initial treatment), limiting imaging to suspected recurrences or treatment failures	7,7	8	2
A one-year follow-up should be considered adequate to rule out a potential frontal recess stenosis	7,6	8	2

**Table 4:** Statements from the Delphi process reaching no consensus

Statements from the Delphi process reaching no consensus	Mean	Median	Outliers
Therapeutic considerations			
Systemic corticosteroids are not recommended for patients with uncomplicated PPT, but may be indicated in patients with intracranial involvement, only in accordance with neurosurgeons	7,1	8	6
The use of endonasal stents in acutely infected scenarios such as PPT shouldn’t be considered a standard choice	7,5	8	5
Frontal sinus obliteration is not necessary in primary cases of PTT, but may be considered in cases where frontal sinus drainage cannot be established, or can be considered when a craniotomy is performed to treat an intracranial collection. Care must be taken to remove all mucosa to prevent mucocele formation.	7,5	8	4

In line with the recommendations outlined by Rosenfeld et al. [9], we adopted a rigorous and transparent approach to the interpretation of consensus outcomes. Specifically, we applied strict thresholds for defining “strong consensus” (mean ≥ 8.00 with no outliers) and “consensus” (mean ≥ 7.00 with no more than one outlier), ensuring that only statements with high levels of agreement and minimal variability were retained. This high-agreement standard, as recommended by Rosenfeld, was purposefully chosen to reduce ambiguity and enhance the clarity and clinical relevance of the resulting statements, especially in areas where practice variation persists. Our methodology emphasizes precision and reliability in expert consensus, providing a robust foundation for harmonizing clinical practice. High-agreement issues disclosed some degree of heterogeneity in corticosteroid prescription, timing of surgery and long-term management of the frontal sinuses, and pointed to direction for further research.

**Fig. 2:**
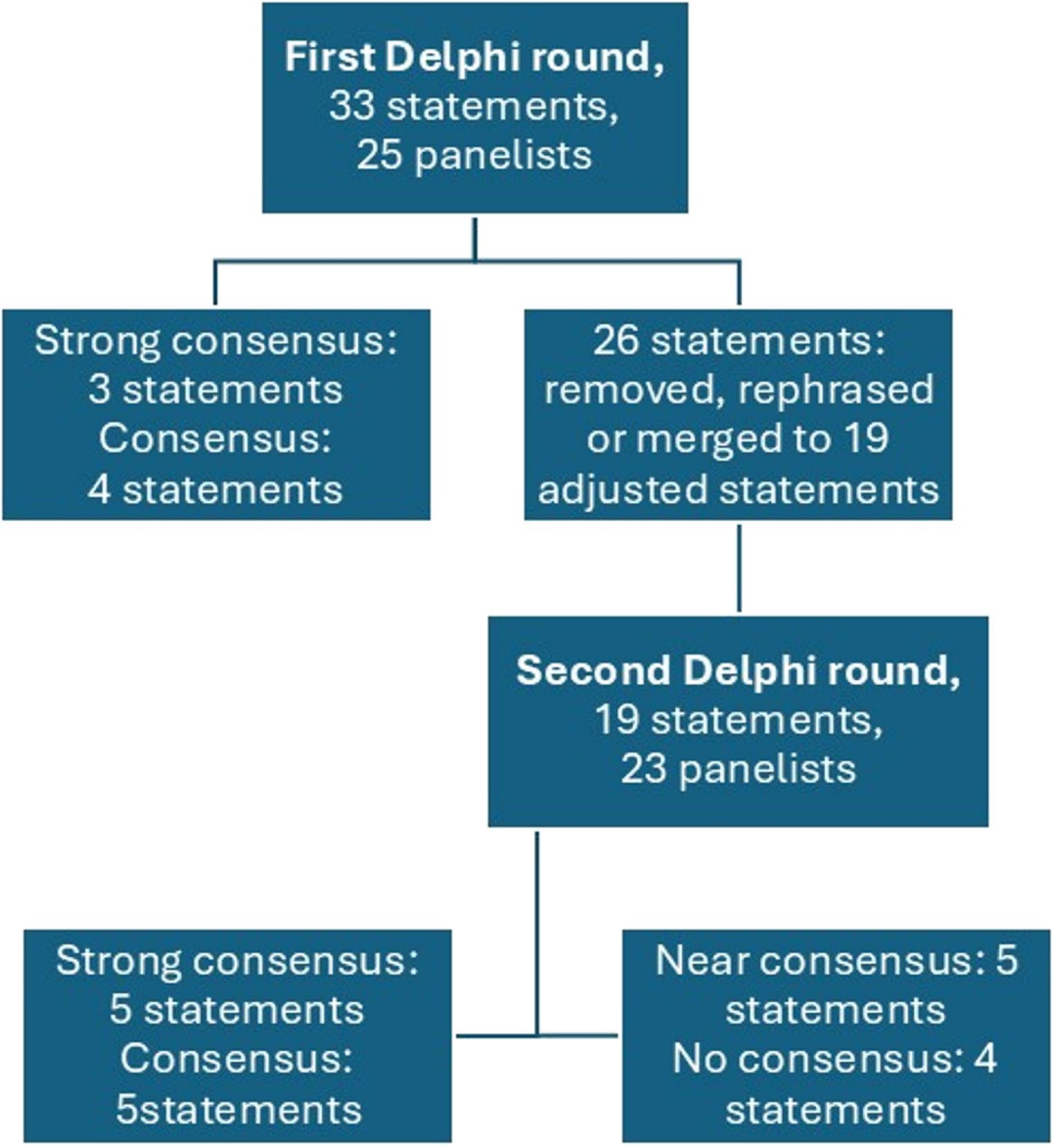
Flowchart and results of the Delphi process

Table 5 gives an overview of the study characteristics and main findings of the included studies of our literature review. The workflow of the Delphi consensus is also shown in Fig. 2.

**Table 5:** Summary of findings of literature review

Authors	Topic	Type	No. of PPT patients	Age group (ped/adult)	Age (mean, years)	Male/Female (%M/%F)	Main findings and conclusions
Nisa et al.	Orbital complications in patients with PPT	SR	42	ped/adult	17,44	67%M/23%F, 10%NR	The main findings are that (a) the majority of orbital involvement in PPT is eyelid edema and preseptal cellulitis, (b) most orbital involvement is cured with the treatment of PPT and does not require additional orbital surgery, and (c) permanent orbital sequelae is rare.
Leong et al.	Outcomes and efficacy of ESS for PPT	SR	29	ped/adult	25	69%M/31%F	Uncomplicated PPT can be managed successfully with ESS, although the revision rate for ESS is not low (10%)
Koltsidopoulos	Assess rate of intracranial involvement in pediatric PPT	SR	93	ped	11,94	70%M/30%F	On the basis of the available literature, it seems that young children and adolescents with PPT are at high risk for intracranial complications (72%). Epidural empyema is the most prevalent 47% and 23% had more than one intracranial complication.
Rohde et al.	Review of PPT in both pediatric and adult population	SR	321	ped/adult	29,1	70,4%M/29,6%F	No specific recommendations other than high index of suspicion for intracranial complications, multidisciplinary and individualized approach
Sideris et al.	Review of PPT with intracranial complications in adults	SR	125	adult	45.14	75,2%M/24,8%	Intracranial complications are more common in adult males in the fourth decade of their lives. Diabetes mellitus and immunosuppressive conditions are independent prognostic factors for the development of intracranial complications
McNeil et al.	Streptococcus anginosus infections in children	LRS	15	ped	11.1	66.7%M/33,3%F	Patients with intracranial infection and/or PPT were more likely to be admitted to the intensive care unit and to have venous thrombosis, had higher C-reactive protein values and longer duration of hospitalization (compared to patients with mastoiditis or orbital abscess). A significant increase in disease rate was noted for PPT caused by Str. anginosus group, from 2011 to 2018.
Nallani et al.	Odontogenic disease in adults with PPT	LRS	17	adult	61,9	58,8%M/41,2%F	Upon dentist review, 16 (94%) had various odontogenic pathology visible on their computed tomography scans. Clinicians should assess the oral cavity and dental health of patients with PPT.
Nguyen et al.	Outcomes of surgical procedures for PPT	LRS	31	ped/adult	42	55%M/45%F	With early diagnosis and mild disease, FESS is sufficient to prevent recurrence of PPT but chronic sinusitis may continue to occur if frontal sinus outflow track is not well opened.
Klivitsky et al.	Retrospective chart review of PPT in children	LRS	10	ped	12,5	60%M/40%F	Of the ten children, 70% had intracranial complications. All children underwent surgical treatment, with 40% neurosurgical drainage. All children had full recovery.
Smiljkovic et al.	Intracranial complications of sinusitis	LRS	27	ped	12	65%M/35%F (all patients)	All data are about the entire cohort of intracranial complications of sinusitis, with no specific information about the PPT patients. The data show neurologic sequelae following intracranial complications in 24% of the patients, these are associated with the presence of cerebritis and more extensive intracranial involvement on neuroimaging suggesting that delayed diagnosis is a major contributor to adverse outcomes.

SR: systematic review, LRS: large retrospective series, NA: not applicable, PPT: pott’s puffy tumor, Ped: pediatric, NR: not reported

## Discussion

The purpose of this CCS is to systematically assess diagnostic and therapeutic approaches of patients with PPT, using the best available evidence and expertise of the panel.

Furthermore, we hope that items that did not reach consensus may serve as areas for future research.

### Diagnostic considerations

In patients with suspected PPT, imaging is necessary to confirm the diagnosis, establish the extent of sinus pathology, and look for any intracranial complications. There is no evidence on the timing of imaging and preferred imaging modality. CT typically shows opacified frontal sinuses with swelling over the overlying scalp and may demonstrate a subperiosteal abscess and intracranial complications. Additionally, MR imaging may detect more subtle intracranial involvement, such as enhancement of the dura, extra-axial fluid collections, and/or cerebritis [10]. Also, dural sinus thrombosis (DST) and bone marrow signal changes can be more adequately detected on MR images [11]. It might be worth noting here, as this complex issue was considered beyond the scope of the present consensus, that at present anticoagulant therapy is widely used as a first-line treatment for DST, with thrombolysis reserved for deteriorating cases [12]. Yet, the evidence on this condition is overall scarce, and nearly non-existent for cases of PPT.

A strong consensus was reached on the statement that in patients with suspected PPT, imaging should be obtained urgently. Due to the high incidence of intracranial complications, imaging of the brain is mandatory. A strong consensus was also reached on the statement that a brain MRI might complement CT scan findings because it can show intracranial complications that are not detectable by a CT scan. Delayed diagnosis of intracranial complications has been described in many cases in the literature, where intracranial spread has been missed on CT images and only became apparent after MR imaging [13–16]. This should especially be considered in resource-poor environments, where access to MRI might be limited [17]. Table 6 briefly compares CT and MRI as imaging options for PPT summarizing the merits, demerits and availability of the two for various clinical setups.

**Table 6:** – CT vs. MRI for pott’s puffy tumor: diagnostic strengths and limitations

CT	MRI
Excellent visualization of bony structures and sinus opacification	Superior for evaluating intracranial complications (e.g., cerebritis, abscess, dural sinus thrombosis)
Can detect subperiosteal abscesses and bone erosion	Can identify subtle brain involvement missed on CT
Lower cost and wider availability compared to MRI	Limited availability in some centers, especially in low-resource settings
Faster acquisition time, useful in the emergency setting	No ionizing radiation, preferable in children

CT, computed tomography; MRI, magnetic resonance imaging

Other diagnostic tests that patients with PPT should have are complete blood count with differential and microbiology samples from the infected sinus or from the abscess to tailor the antibiotic treatment. As some PPT cases represent a complication of odontogenic sinusitis, such a differential etiology should be considered and, in suspect cases, evaluated with a dental provider [1, 3, 18].

### General treatment principles

General treatment principles of PPT were an area of overall good consensus, with the notable exception of the role of systemic corticosteroids. Medical treatment was deemed to be the cornerstone in the multidisciplinary approach (MA) to PPT, as highlighted by the strong consensus statement emphasizing the role of a team including otolaryngologists, infectious disease specialists, neurosurgeons, pediatricians, and intensivists. This approach ensures comprehensive decision-making, management, and follow-up. The necessity of this collaborative framework is supported in literature [5, 19, 20].

A strong consensus also emerged regarding the use of broad-spectrum antibiotics tailored to local resistance patterns and capable of penetrating the blood-brain barrier in cases with intracranial involvement. This is supported by findings from Nguyen et al., where resistant infections following inadequate prior treatment due to drug allergies were common, often requiring empiric coverage of gram-positive organisms and anaerobes until culture results guide targeted therapy [21]. Similarly, Klivitsky et al. emphasized the importance of microbiological studies to refine antibiotic regimens, noting a predominant presence of the Streptococcus group in their cohort [2].

Regarding intravenous (IV) antibiotics, a consensus was achieved that hospitalization is mandatory for the initial phase of treatment. Moreover, there was a consensus recommendation to customize antibiotic treatment based on culture results and its duration by considering the overall clinical picture. This is because factors like the pathogen, osteomyelitis grade, and potential intracranial complications should all be taken into account, ideally in collaboration with an infectious disease specialist. These points align with Leong et al., who noted that long-term IV therapy with optimal bioavailability can be crucial for disease resolution, even after limited surgical treatment without external drainage [8]. Duration of IV therapy should be individualized based on the degree of osteomyelitis and intracranial involvement, a point strongly emphasized by Sideris et al., who documented better outcomes with prolonged and tailored antibiotic courses [6]. In addition to clinical features and the severity of presentation, antimicrobial treatment decisions should align with established principles of antibiotic stewardship [22]. Empiric therapy should be appropriately targeted based on the most likely pathogens and local resistance patterns, while avoiding the routine use of broad-spectrum or reserve antibiotics unless clearly justified. Agents such as carbapenems or advanced-generation cephalosporins should be reserved for cases with documented multidrug-resistant organisms or treatment failure. Whenever feasible, culture-directed therapy is recommended, and de-escalation should follow once microbiological data become available. This approach minimizes the development of antimicrobial resistance, reduces potential adverse effects, and supports the long-term effectiveness of essential antibiotic agents.

While general principles for antibiotic therapy were received with good to excellent consensus, the use of corticosteroids was definitely more debated, with the respective statement not reaching any consensus. This mirrors the fragmented data available and the strong heterogeneity in PPT management between different specialists and countries. The statement with the lowest score or agreement was regarding the role of systemic corticosteroids in PPT treatment. Two main points of debate for corticosteroids emerged. On the one hand, the panel felt that the use of corticosteroids has no proven role in the course of uncomplicated PPT, as has also been debated in multiple papers on orbital cellulitis [23–25]. Additional arguments against the routine use of systemic corticosteroids are suppression of the immune system and possible worsening of infection [24]. Furthermore, the panel felt that the use of corticosteroids in managing intracranial complications, which is indeed indicated in selected cases, should be managed by neurosurgeons and tailored to the specific patient and degree of intracranial involvement [26]. The wide variation in corticosteroid use demonstrated in this Delphi process mirrors the uncertainty found with other disease processes, including brain abscess and orbital cellulitis, underscoring the need for disease-specific treatment trials.

In conclusion, the panel judged that the medical treatment of PPT necessitates a MA with individualized therapy plans based on culture results and clinical severity. This is also reflected in guidelines about the management of intracranial complications of sinusitis in children, where strong emphasis has been placed on the use of empiric broad-spectrum antibiotics, targeted rationalization of antibiotic treatment, and where corticosteroid treatment was primarily described for the treatment of cerebral oedema [27].

### Surgical treatment options

The panel reached strong consensus that the initial surgical approach for treatment of PPT can include incision and drainage of the subperiosteal abscess, endoscopic sinus surgery, or trephination, or a combination of these approaches and will depend on the patients age, abscess location, surgeon’s preference and experience, and locally available instrumentation and resources. In a literature review of 92 children and adolescents with PPT, 45% had an external incision and drainage of the subperiosteal abscess, and 50% had an endoscopic procedure, of which about half were in combination with an open procedure [19]. In a literature review of 321 children and adults with PPT, more than 50% of patients had an endoscopic procedure in combination with an open approach or alone, with approximately 32% undergoing an external drainage procedure. In addition, the determination of the optimal approach depended on clinical features, including age and the presence of intracranial involvement on presentation [3]. In a 2017 review of 29 patients, 59% had an endoscopic sinus surgery procedure, which was shown to be feasible in patients with a median age of 25 years (range 9–86 years) due to the availability of powered angled instruments, high-definition video, and image guidance system [8].

There was consensus on the goals of surgery: to drain both the subperiosteal abscess and the sinuses - including the frontal sinus - of purulence. This is in line with findings from the two large literature reviews [3, 19]. Both studies also discuss the goal of surgery to include the debridement or removal of osteomyelitic bone or foci, which was historically addressed using an external approach. However, with the advent of endoscopic sinus surgery, both studies highlight the safety and efficacy of an endoscopic approach to establish natural drainage of the frontal sinus outflow tract in management alone or in combination with an external approach. Rhode also highlights variables that may preclude an endoscopic approach, including profound mucosal edema and loss of anatomical landmarks, which may require initial external drainage, followed by second-stage endoscopic sinus surgery [3]. The panel also reached the consensus that the placement of an external drain should be considered after external drainage/trephination in case of insufficient endonasal drainage of the frontal sinus, also recommended by Koltsidopoulos et al. to avoid abscess recollection [19]. Near consensus was reached about the goal of frontal sinus drainage, to clear purulent material and to provide a drainage pathway in an atraumatic fashion. Comments were predominantly regarding what defines “atraumatic,” as even endonasal frontal sinus surgery procedures, although minimally invasive, can induce synechiae and complications. Another panelist questioned which was more atraumatic: “to perform a direct opening of the frontal sinus through external approach or to perform a Draf procedure with associated risks of iatrogenic stenosis of the frontonasal duct?” Both are important points and highlight the complexity in surgical decision-making.

There was near consensus from the panel that the subperiosteal abscess of the frontal bone might require an external approach for drainage in selected cases. Though a recent SR of 184 patients, with an average age of 12 years, shows that the majority of the authors included in the review opted for an external approach for draining the subperiosteal abscesses [28]. For patients with intracranial complications, craniotomy was used in isolation or in combination with external drainage of the frontal sinuses and/or with endonasal surgery [28]. Multiple approaches may be required simultaneously to address the intracranial abscess, and the goal specific to the frontal sinus will be to reestablish adequate frontoethmoidal drainage.

A consensus was also not reached on the timing of surgery. The standard for the management of PPT includes antibiotics and operative drainage of infection for which the treatment is not only “effective” but also “rapid,” “early,” and “prompt,” though many large reviews do not include a time frame within which to go to surgery [3, 19, 20]. There was near consensus that patients with PPT without intracranial complications who are not improving with antibiotic treatment should undergo surgical drainage, ideally within 24 h. Comments from panelists included a lack of evidence in terms of providing a time frame for surgical intervention, as well as the need to consider the nuances in each clinical context that can play a role in surgical decision-making.

More advanced or radical approaches to frontal sinus long-term management were indeed the most critical part of the surgical management consensus. Namely, the panel did not reach a consensus in supporting the use of endonasal stents in acutely infected scenarios such as PPT. There is published data, including an expert consensus and a meta-analysis for the indications for steroid-eluting stents for chronic rhinosinusitis after endoscopic sinus surgery, but there is no published data on the use of steroid-eluting stents in the setting of acute rhinosinusitis [29, 30]. There is very little published data regarding long-term sinus stenting for chronic frontal sinus disease after endoscopic sinus surgery, and this is a subject of debate [31–34]. There is currently no data for this intervention in patients with acute rhinosinusitis after surgery. Similarly, the lack of evidence and heterogeneity in surgical management between different surgeons and international centres, led to a lack of consensus for the role of frontal sinus obliteration.

From a general surgical perspective, data gained from this CCS suggest that comparative, preferably multicenter or registry-based, investigations are required to assess the appropriateness and long-term results of various means of frontal sinus drainage.

### Follow-up and outcomes

Not all included studies reported on outcomes of the patients. Leong et al. reported that 10.3% of the patients required revision surgery during the follow-up period [8]. Nallani et al. on the other hand, reported that none of the patients required revision surgery; however, the duration of follow-up was not mentioned [1]. McNeil et al. reported that 6.7% of the patients had neurological deficits at last follow-up, and 13.3% were admitted for recurrence of infection during follow-up [35]. Smiljkovic et al. found long-term neurological sequelae of intracranial complications of sinusitis in 24% of patients, but it was not specified which were accompanied by PPT [5]. Klivitsky et al. reported a full recovery in all patients despite 70% having intracranial involvement [2]. Surprisingly, two of the included reviews did not report on outcomes, recurrences, revision surgery, or long-term sequelae [3, 19].

The panel reached a consensus that in surgically treated PPT patients with endoscopic or radiological evidence of inadequate sinus drainage on follow-up, an elective ESS should be considered in case of persisting symptoms. However, following the two Delphi rounds, our statements on short- and long-term follow-up did not reach a consensus. The reasons given by the expert panel were: the follow-up will be dependent on the age of the patient, the type of surgical treatment, and the presence of intracranial involvement. Endoscopically treated patients may require close and long-term follow-up with nasal endoscopy to rule out frontal recess stenosis, which may appear even after more than one year. Mucocele formation should be considered as a long-term complication after frontal sinus surgery, especially after obliteration [36]. There was no agreement or evidence on the role of imaging during follow-up. Imaging during follow-up is case-dependent and may be assessed according to symptom burden and severity of initial disease and eventual intracranial complications. Taking all considerations into account, we could emphasize that long-term follow-up is necessary, especially in children, to monitor for any long-term complications. Management of follow-up can be age-dependent, correlate with severity of disease, and presence or absence of complications with lead role by otolaryngologists, pediatricians, or neurosurgeons, respectively.

### Limitations and directions for future research

The low quality of the currently available evidence on this topic is one of the most important limitations of this CCS. Most of the data are based on retrospective studies, case series and case reports, or (systematic) reviews of these cases and case series. Given the rarity of the disease, prospective studies will have to be conducted in a multidisciplinary or even international manner to gain enough cases. The organization and administration of such studies come with barriers, including legal and privacy regulations. However, we are aiming and already working towards building an international registry to prospectively collect data on greater numbers of PPT patients. In this way, we could monitor the outcomes of different treatment strategies and gain insight into the epidemiology and microbiology. Such a prospective registry might help first by standardizing reporting of PPT cases, possibly through an ad hoc classification, and then using such data and classification for standardizing approaches and follow-up (methods and length) according to disease extension, location, complications, etiology, and age groups. Such an initiative would not only enable broader epidemiological insights and support evidence-based refinements of diagnostic and therapeutic strategies, but could also gain insight in local and regional differences in care and the differences in cost, access and availability of care (for example MRI, specialized surgical equipment and skills). Other limitations include a lack of synchronous discussion of these statements among our global group. This may have better clarified or added depth to the current statements. Finally, including infectious disease specialists and neurosurgeons in the study group would have also added perspective to the findings.

Though we strived for worldwide representation, and even though the panel was internationally diverse, an inherent participation bias, particularly from under-represented regions, in this Delphi should be taken into account. Analogously, the review of the literature was conducted and included articles written in English, Italian, German, French, and Spanish, thereby excluding potentially relevant studies published in other languages.

## Conclusions

Literature about the diagnosis and management of patients with PPT is limited to retrospective case series and reviews. Performing two Delphi rounds, we have established areas of strong consensus and consensus, aiming to improve diagnosis and care for patients with PPT. Key aspects that reached consensus were the important role of imaging, the need for a high index of suspicion for intracranial complications and adequate imaging of the brain, and the need for a MA. The treatment should consist of intravenous broad-spectrum antibiotics and surgery. Although the goal of surgery is an area of consensus -to drain both the subperiosteal abscess and the sinuses, including the frontal sinus- there was no consensus about the timing of surgery, surgical techniques, and postoperative placement of stents.

This consensus, while the first of its kind to focus on Pott’s puffy tumor, offers much-needed clinical guidance to harmonize diagnostic, surgical, and multidisciplinary care across pediatric and adult patients.

## Supplementary Information

Below is the link to the electronic supplementary material.

ESM 1 (XLSX 12.2 KB)

## References

[1] Nallani R, Wichova H, McAroy JL, Chiu AG, Villwock JA (2021) Incidence of odontogenic disease in patients with Pott’s puffy tumor. J Oral Maxillofac Surg 79:389–397. 10.1016/j.joms.2020.08.001 32890475 10.1016/j.joms.2020.08.001

[2] Klivitsky A, Erps A, Regev A, Ashkenazi-Hoffnung L, Pratt LT, Grisaru-Soen G (2023) Pott’s puffy tumor in pediatric patients: case series and literature review. Pediatr Infect Dis J 42:851–856. 10.1097/INF.0000000000004026 37406183 10.1097/INF.0000000000004026

[3] Rohde RL, North LM, Murray M, Khalili S, Poetker DM (2022) Pott’s puffy tumor: a comprehensive review of the literature. Am J Otolaryngol 43:103529. 10.1016/j.amjoto.2022.103529 35700606 10.1016/j.amjoto.2022.103529

[4] Fokkens WJ, Lund VJ, Hopkins C et al (2020) European position paper on rhinosinusitis and nasal polyps 2020. Rhinology 1–464. 10.4193/Rhin20.600 10.4193/Rhin20.600 32077450

[5] Smiljkovic M, Tat J, Richardson SE et al (2024) A 20-year study of intracranial pyogenic complications of sinusitis in children. Pediatr Infect Dis J 43:91–96. 10.1097/INF.0000000000004140 37851970 10.1097/INF.0000000000004140

[6] Sideris G, Davoutis E, Panagoulis E, Maragkoudakis P, Nikolopoulos T, Delides A (2023) A systematic review of intracranial complications in adults with Pott puffy tumor over four decades. Brain Sci 13:587. 10.3390/brainsci13040587 37190552 10.3390/brainsci13040587 PMC10137007

[7] Flamm ES (1992) Percivall Pott: an 18th century neurosurgeon. J Neurosurg 76:319–326. 10.3171/jns.1992.76.2.0319 1730964 10.3171/jns.1992.76.2.0319

[8] Leong SC (2017) Minimally invasive surgery for pott’s puffy tumor: is it time for a paradigm shift in managing a 250-year-old problem? Ann Otol Rhinol Laryngol 126:433–437. 10.1177/0003489417698497 28376662 10.1177/0003489417698497

[9] Rosenfeld RM, Nnacheta LC, Corrigan MD (2015) Clinical consensus statement development manual. Otolaryngol Head Neck Surg 153(Suppl 2):S1–S14. 10.1177/0194599815601394 26527615 10.1177/0194599815601394

[10] Deng F, Gaillard F (2008) Pott puffy tumour. Radiopaedia Org. 10.53347/rid-4895

[11] Blumfield E, Misra M (2011) Pott’s puffy tumor, intracranial, and orbital complications as the initial presentation of sinusitis in healthy adolescents, a case series. Emerg Radiol 18:203–210. 10.1007/s10140-010-0934-3 21380513 10.1007/s10140-010-0934-3

[12] Ciccone A, Canhão P, Falcão F, Ferro JM, Sterzi R (2004) Thrombolysis for cerebral vein and dural sinus thrombosis. Cochrane Database Syst Rev (1):CD003693. 10.1002/14651858.CD003693.pub2 10.1002/14651858.CD003693.pub2 PMC8725788 14974030

[13] Ketenci I, Unlü Y, Tucer B, Vural A (2011) The Pott’s puffy tumor: a dangerous sign for intracranial complications. Eur Arch Otorhinolaryngol 268:1755–1763. 10.1007/s00405-011-1660-5 21660452 10.1007/s00405-011-1660-5

[14] Olmaz B, Cingoz M, Akdogan E, Kandemirli SG (2019) Correlation of imaging and intraoperative findings in pott’s puffy tumour. Scott Med J 64:25–29. 10.1177/0036933018803787 30293486 10.1177/0036933018803787

[15] Sikka A, Bobby A, Gali R (2024) Beyond the sinus: unmasking pott’s puffy tumor through imaging. Cureus 16:e73000. 10.7759/cureus.73000 39498420 10.7759/cureus.73000 PMC11534264

[16] Bhalla V, Khan N, Isles M (2016) Pott’s puffy tumour: the usefulness of MRI in complicated sinusitis. J Surg Case Rep 2016:rjw038. 10.1093/jscr/rjw038 27001196 10.1093/jscr/rjw038 PMC4800463

[17] Ogbole GI, Adeyomoye AO, Badu-Peprah A, Mensah Y, Nzeh DA (2018) Survey of magnetic resonance imaging availability in West Africa. Pan Afr Med J 30:240. 10.11604/pamj.2018.30.240.14000 30574259 10.11604/pamj.2018.30.240.14000 PMC6295297

[18] Craig JR, Cheema AJ, Dunn RT, Vemuri S, Peterson EL (2022) Extrasinus complications from odontogenic sinusitis: a systematic review. Otolaryngol Head Neck Surg 166:623–632. 10.1177/01945998211026268 34253072 10.1177/01945998211026268

[19] Koltsidopoulos P, Papageorgiou E, Skoulakis C (2020) Pott’s puffy tumor in children: A review of the literature. Laryngoscope 130:225–231. 10.1002/lary.27757 30570150 10.1002/lary.27757

[20] Kühn JP, Linsler S, Nourkami-Tutdibi N et al (2022) Pott’s puffy tumor: a need for interdisciplinary diagnosis and treatment. HNO 70(Suppl 1):8–13. 10.1007/s00106-021-01134-w 35072731 10.1007/s00106-021-01134-w PMC8837573

[21] Nguyen DK, Idicula W, Nguyen T, Demke J, Cordero J, Dundar Y (2023) Pott’s puffy: first shot is the best shot. J Craniofac Surg 34:1522–1525. 10.1097/SCS.0000000000009451 37307535 10.1097/SCS.0000000000009451

[22] Nathwani D, Varghese D, Stephens J, Ansari W, Martin S, Charbonneau C (2019) Value of hospital antimicrobial stewardship programs [ASPs]: a systematic review. Antimicrob Resist Infect Control 8:35. 10.1186/s13756-019-0471-0 30805182 10.1186/s13756-019-0471-0 PMC6373132

[23] Gill PJ, Mahant S, Hall M et al (2022) Association between corticosteroids and outcomes in children hospitalized with orbital cellulitis. Hosp Pediatr 12:70–89. 10.1542/hpeds.2021-005910 34877598 10.1542/hpeds.2021-005910

[24] Kornelsen E, Mahant S, Parkin P et al (2021) Corticosteroids for periorbital and orbital cellulitis. Cochrane Database Syst Rev (4):CD013535. 10.1002/14651858.CD013535.pub2 10.1002/14651858.CD013535.pub2 PMC8092453 33908631

[25] Kim BY, Bae JH (2022) Role of systemic corticosteroids in orbital cellulitis: a meta-analysis and literature review. Braz J Otorhinolaryngol 88:257–262. 10.1016/j.bjorl.2021.02.003 33722520 10.1016/j.bjorl.2021.02.003 PMC9422736

[26] Gundamraj S, Hasbun R (2020) The use of adjunctive steroids in central nervous infections. Front Cell Infect Microbiol 10:592017. 10.3389/fcimb.2020.592017 33330135 10.3389/fcimb.2020.592017 PMC7719626

[27] Raineau M, Crowe AM, Beccaria K et al (2024) Pediatric intracranial empyema complicating otogenic and sinogenic infection. Int J Pediatr Otorhinolaryngol 177:111860. 10.1016/j.ijporl.2024.111860 38224655 10.1016/j.ijporl.2024.111860

[28] Daloiso A, Mondello T, Boaria F et al (2024) Pott’s puffy tumor in young age: A systematic review and our experience. J Clin Med 13. 10.3390/jcm13216428 10.3390/jcm13216428 PMC11546441 39518567

[29] Goshtasbi K, Abouzari M, Abiri A et al (2019) Efficacy of steroid-eluting stents in management of chronic rhinosinusitis after endoscopic sinus surgery: updated meta-analysis. Int Forum Allergy Rhinol 9:1443–1450. 10.1002/alr.22443 31539461 10.1002/alr.22443 PMC6901756

[30] Lee VS, Patel P, O’Brien D et al (2022) Indications for absorbable steroid-eluting sinus implants: viewpoint via the Delphi method. Int Forum Allergy Rhinol 12:1225–1231. 10.1002/alr.23044 35730163 10.1002/alr.23044 PMC10108565

[31] Hunter B, Silva S, Youngs R, Saeed A, Varadarajan V (2010) Long-term stenting for chronic frontal sinus disease: case series and literature review. J Laryngol Otol 124:1216–1222. 10.1017/S0022215110001052 20482950 10.1017/S0022215110001052

[32] Khan MA, Alshareef WA, Marglani OA, Herzallah IR (2020) Outcome and complications of frontal sinus stenting: A case presentation and literature review. Case Rep Otolaryngol 2020:8885870. 10.1155/2020/8885870 10.1155/2020/8885870 PMC7471784 32908758

[33] Orlandi RR, Knight J (2009) Prolonged stenting of the frontal sinus. Laryngoscope 119:190–192. 10.1002/lary.20081 19117282 10.1002/lary.20081

[34] Weber R, Mai R, Hosemann W, Draf W, Toffel P (2000) The success of 6-month stenting in endonasal frontal sinus surgery. Ear Nose Throat J 79:930–932934937 11191431

[35] McNeil JC, Dunn JJ, Kaplan SL, Vallejo JG (2020) Complications of otitis media and sinusitis caused by *Streptococcus anginosus* group organisms in children. Pediatr Infect Dis J 39:108–113. 10.1097/INF.0000000000002514 31738321 10.1097/INF.0000000000002514

[36] Hansen FS, van der Poel NA, Freling NJM, Fokkens WJ (2018) Mucocele formation after frontal sinus obliteration. Rhinology 56:106–110. 10.4193/Rhin17.187 29396959 10.4193/Rhin17.187

